# Fitting Health Financing Reforms to Context: Examining the Evolution of Results-Based Financing Models and the Slow National Scale-Up in Uganda (2003-2015)

**DOI:** 10.1080/16549716.2021.1919393

**Published:** 2021-05-11

**Authors:** Aloysius Ssennyonjo, Elizabeth Ekirapa–Kiracho, Timothy Musila, Freddie Ssengooba

**Affiliations:** a Department of Health Policy Planning and Management, School of Public Health, Makerere University, Kampala, Uganda; b Ministry of Health, Kampala, Uganda

**Keywords:** Results-based financing schemes, diffusion of innovations, scale-up, health financing reforms, UHC, Uganda

## Abstract

**Background**: Results-based financing has been promoted as an innovative mechanism to improve the performance of health systems in achieving universal health coverage. Several results-based financing models were implemented in Uganda between 2003 and 2015 but with limited national scale-up.

**Objective**: This paper examines the evolution of results-based financing models and the reasons for the slow national adoption and implementation in Uganda.

**Methods**: This was a qualitative study based on document review and key informant interviews. The models were compared to show modifications overtime. The reasons for the slow national scale-up were analyzed using variables from the Diffusion of Innovations Theory.

**Results**: This study covered seven schemes implemented in the Ugandan health sector between 2003 and 2015. The models evolved in several aspects: 1) donor reliance with fundholding and purchasing delegated to non-state organizations; 2) establishment of ad-hoc structures for learning; 3) recent involvement of the government agencies in verification processes; 4) Involvement of public providers, and 5) expansion of services purchased from the national minimum health-care package. The main reasons for slow national adoption were the perceived complexity and incompatibility with public sector systems. The early phases comprised barriers to public sector reforms. However, recent adjustments to the schemes have enabled greater involvement of public providers and government stewardship. Stakeholders also reported progressive learning across projects and time.

**Conclusion**: Overall, the study findings show scheme actors’ deliberate efforts to adapt their models to the Ugandan health system and public sector context. Results-based financing is a complex intervention that takes time for the capacity to be built among vital actors. Progressive re-designing of models enhances fitness to the health systems context. From this study, we advise that Uganda and similar countries should undertake deliberate efforts to customize such models to the capacity and institutional architecture of their health systems.

## Background

### Background

By definition, results-based financing (RBF) refers to “a cash payment or non-monetary transfer made to a national or sub-national government, manager, provider, payer or consumer of health services after attainment and verification of predefined results” [1]. RBF is an umbrella term for several approaches that involve linking payments to the achievement of quantitative or qualitative indicators. RBF has been used synonymously with pay for performance (P4P), performance-based payment and performance-based incentives (PBI) [2].

Over the last two decades, RBF mechanisms have emerged as a means to improve the performance of health systems (and recently) to support progress towards universal health coverage (UHC) [3,4]. The potential mechanisms through which RBF strengthens health systems’ performance include the strategic use of health financing to pay for results, incentivizing the provision of desired health services, promoting improvements in health information systems and building capacity for governance and regulation [5]. These observations have stimulated increased global interest in scaling-up RBF and integrating it into national health systems [6]. However, RBF is a complex intervention to scale-up into national health financing systems. Although all RBF projects link financial incentives to results (outputs or outcomes), they have unique features which may influence their implementation even within the same national health system contexts [5,7]. Bertone & Meessen [8] highlighted the complex pathway to the institutionalization of RBF. These include changes in the institutions, enforcement mechanisms, property rights, incentives, interactions between extrinsic and intrinsic sources of motivation for the workforce, and organizational behaviours towards performance management. Witters et al. [3] elaborated a framework for monitoring and evaluating RBF and health systems interaction. The authors underscored the dynamic interactions among context, policy formulation, design, implementation, and systems effects. These interactions demand active exploration of how to fit this complex intervention into the national health systems.

In 2014, the Alliance for Health Policy and Systems Research (AHPSR) at the World Health Organisation (WHO) issued a call for research on facilitators and barriers to transitioning RBF initiatives in low- and middle-income countries from pilot projects to national-wide health systems [9,10]. This multicountry study examined how RBF innovations moved from projects into being fully integrated into national health systems [9,11]. Uganda was one of the case studies. Relative to her neighbours (Rwanda and Burundi), the scale-up of RBF in Uganda was considered slow and dismal despite several generations of standalone schemes [6].

This paper examines the evolutions in RBF models implemented in Uganda between 2003 and 2015. We recognize that although some information is available on the design and institutional arrangements of several RBF schemes that were implemented in Uganda between 2003 and 2015, there has not been any systematic and comprehensive examination of their design attributes. The second objective of this paper is to examine why these models took so long to be scaled-up at the national level. Scale-up is defined narrowly as the adoption of RBF as a national program. Evidence indicates that the design elements of the RBF model can hinder scale-up [6,7].

### Overview of the Ugandan health system

The Ugandan health system is composed of both public and private sectors in terms of ownership of infrastructure and the delivery of health services [12]. In terms of health financing, Uganda’s Government introduced a ‘free’ health-care policy in public facilities after abolishing user fees in 2001. However, the private sector uses a fee-for-service payment model [13]. Out-of-pocket payments (OOPs) are still high at a proportion of 43% of total health expenditure in 2015 [14]. RBF pilots have been designed mostly by donors and their fund-holders to reduce user fees and out-of-pocket payments through vouchers and subsidies to private health providers. Donors who provide both ‘on-budget’ and ‘off-budget’ support to the health sector have increasingly adopted RBF as a precondition for their support. Examples include the Global Fund to Fight AIDS, Malaria and Tuberculosis, and the USA President’s Emergency Plan for AIDS Relief (PEPFAR) [15].

Like many developing countries, the functionality of health facilities in Uganda is sub-optimal [16] due to limited management capacity, inadequate funding, poor infrastructure and workforce shortages. Stock-outs of medicines and other health supplies are still a challenge despite government efforts to curb this problem. These upstream issues undermine the health system’s performance despite innovations such as RBF introduced to enhance health providers’ performance.

## Methods

### Study design and data collection approaches

This study adopted a qualitative research approach to track the evolution of RBF in Uganda between 2003 and 2015 when data collection took place. We used key informant interviews, participation in national RBF dialogues and consultation meetings, and review of RBF related documents to analyze the different RBF designs and to determine changes in RBF models overtime and generate plausible explanations for the slow RBF scale-up process in Uganda.

Documents reviewed included reports of national RBF consultation meetings, grant applications, special studies, scientific publications, pilot project implementation and evaluation reports and national strategic documents about financing and purchasing. These reports provided background context and outlined issues of concern as well as justifications for using RBF approaches in the health interventions in Uganda. We conducted 39 Key informant interviews with RBF stakeholders – covering implementers, project staff, health facility managers, national-level policy-makers from government ministries, academic and private sectors, district policy-makers and development partners (see Table 1). These respondents were identified from the literature and national consultation meetings based on their RBF implementation and health systems management roles in Uganda. The research team is well embedded in the health system context, and this enabled easy identification and contacting of the respondents. The respondents were approached through email and phone calls; consent was reaffirmed in face-to-face interviews conducted at workplaces. Participant observations were conducted during national consultation events held in February 2015 [17]. Interviews lasted on average of 30 minutes.

**Table 1.** Table showing categories of respondents

Category	Sub category	Number of respondents
Implementers/project staff		10
Health facility managers		7
National level policy-makers	MOH	5
	Academics	3
	Ministry of Finance	1
	Private sector	1
District policy-makers	District health Officers/DHT	5
Development partners		7
TOTAL		39

The interviews were audio-recorded and transcribed verbatim. Although the interviews covered a broad range of issues surrounding RBF in Uganda, the analysis in this paper draws on the questions related to the evolution of the designs of RBF models and reasons for the slow RBF integration in the national health systems. Reasons for the adjustments were explored, especially during the 2015 RBF consultation meeting [17] and during interviews with key actors in the RBF processes.

## Analytical framework

### RBF models and their design elements

RBF models are characterized by 1) use financial or non-financial incentives used by a principal (e.g. donor or government) to influence the performance of the agent (e.g. service providers or communities); 2) delivery of results by the agent; 3) provision of reward after verification of performance goals – usually done by a third party; 4) Contractual arrangements that embody the performance targets and rewards. In practice, the RBF models may be designed to function as extra efforts on top of the traditional fee-for-service or input-based financing [1]. The variations in RBF models are contingent on the diverse ways the RBF design elements get operationalized. According to Meessen et al. [11], these design elements include the following: 1) management and governance structures with a focus on the contractual separation of functions of funder holding, purchasing, regulation and service provision/beneficiary; 2) verification of service volumes, quality and/or outcomes, information and payment systems; 3) Institutional support and readiness; 4) Involvement of demand-side actors and 5) service coverage and population coverage or geographical scope. These design elements formed the domains for comparing the RBF models in Uganda.

### Diffusion of innovations theory

This study considered RBF as a health systems innovation. Thus, the study drew on constructs from the DOI theory to analyze the reasons for the slow national scale-up of RBF in Uganda [18]. The Diffusion of Innovations Theory (DOI) theory advances that the following characteristics of an innovation affect its rate of adoption as elaborated below:

- **Relative advantage** is the extent to which an innovation is perceived as better than the idea it replaces. Parameters include perceptions on economic returns, convenience, and effectiveness of the invention. The greater the perception of relative advantage, the faster the diffusion rate of the innovation irrespective of the findings from objective measures.
- **Compatibility**: The degree to which potential adopters perceive the innovation to be consistent with current expectations, norms, past experiences, and their needs. Innovations compatible with the existing social systems are easier to adopt as those incompatible require the prior establishment of new value systems.
- **Complexity** is the extent to which an idea/innovation is considered complicated and hence difficult to understand and use. New ideas that are easier to understand are adopted more easily than new reforms or components deemed problematic.
- **Trialability** is the degree to which an innovation can be experimented on a small scale through pilot and other demonstration projects. The trialability of an innovation improves the likelihood of adoption.
- **Observability**: The degree to which others can see the results of an innovation. The more visible the results of an innovation are to the potential adopters, the higher the likelihood of its being adopted.

## Results

### Changes in RBF models over 2003-2015

This study covered seven RBF schemes implemented in the Ugandan health sector between 2003 and 2015. Four of these were supply-side, while three were demand-side schemes. The supply-side schemes studied included the World Bank Performance-Based Contracting (PBC) Study (2003–2005), the Cordaid Pilot (2009–2016), the NuHealth project (2011–2015) and the Strengthening Decentralisation for Sustainability (SDS) (2011–2016). The three demand-side schemes included the Reproductive Health Voucher Project by the World Bank (2006–2011), the Safe Deliveries Project (SDP) (2009–2011) and the Saving Mothers Giving Life (SMGL) Initiative (2011–2017).

Tables 2 and 3 below elaborate the general design and key actors involved in the examined RBF pilots, as highlighted in the interviews and the documents reviewed [7,19–24].

**Table 2.** General design and key actors in various schemes

Project feature	Duration	General pilot design	Funder	Fund holder	Purchasing agent	Auditing/Verification agents
Supply side schemes						
World Bank Study	2003–2005	Quasi-experimental design, two intervention groups and a control	CIDA, USAID,BTC	World Bank-Washington	World Bank through local government.	Makerere University School of Public Health (MakSPH)
Cordaid project	2009–2015	Interventional design	Cordaid	Cordaid	Jinja Diocese/Cordaid	Cordaid/District health teams (DHTs) and Community-Based Organisations (CBOs)
NuHealth Project	Sept 2011–2015	Quasi-experiment study (RBF & input based financing)	UKAid (formerly DFID)	Health Partners International (HPI) &Montrose International	HPI & Montrose	NU-Health and District health teams (DHTs)
SDS Project	2011–2016	Intervention design	USAID	SDS program	Cardno and other agencies such as (IDI)	SDS + District health teams
Demand side/voucher schemes						
Reproductive Health vouchers Project	July 2006–2011	Intervention study	KfW and the GPOBA-World Bank)	Maries Stopes Uganda (MSU	MSU	MSU PWC as independent verifier.
Safe deliveries Project (SDP)	2009–2011	Quasi-experiment study intervention and control.	Bill and Melinda Gates Foundation and WHO-AHPSR	MakSPH	MaKSPH	MakSPH
Health Baby/SMGL Voucher Project.	2012-2017	Intervention design	SMGL funded by US Global Health(GHI) and partners	SMGL initiative	Baylor-Uganda, IDI,STRIDES for family health, MSU.	Respective agencies

**Table 3.** Changes in geographical scope and service packages across schemes

Project	Population coverage		Service coverage	
	Geographical scope	Populations served	Service packages	Facilities
Supply side schemes				
World Bank Study	118 facilities (68 PNFPs) from five pilot districts distributed in four regions. No change over project life.	All resident within reach of health facilities	Six service priorities (OPD and malaria, immunization, ANC, attended births & Family planning.	Intervention group included PNFP only. Public, Private sectors in control category.
Cordaid project	Initially three districts in east (Jinja, Kamuli & Iganga). Later restricted to Kamuli.	All residents within reach of facilities	Range of services from national package.	Started with PNFP. Extended to public facilities in 2013
NuHealth Project	31 health centres in two regions or 12 northern Uganda districts. No change in scope overtime.	All residents within reach of facilities	Range of services especially maternal and child health services	PNFP only.
SDS	35 districts initially increased to 50 districts in 2015 across the country.	Local governments and Medical bureaux	Performance-based grants to districts and Medical Bureaux incentivise governance and management functions.	In regard to health services, the facilities targeted were those with bias to HIV/AIDS and the PNFP facilities
Demand side/voucher schemes				
Reproductive Health vouchers Project	Evolved from four pilot districts to 20 districts in south western Uganda.	Women for Safe Motherhood (SM) Couples for STI. Poverty grading used to target poorest.	SM services &STI treatment.	PFP and PNFP facilities. Public facilities were referral points
Safe deliveries Project (SDP)	22 health facilities in two districts in Eastern Uganda. No change in scope.	All pregnant women, transport providers used.	MCH and health system strengthening component to deliver obstetric care services.	Public, PFP and PNFP facilities
Health Baby/SMGL Voucher Project.	Four districts in Western Uganda but scaled up to 10 (included six more districts in Northern Uganda)	All pregnant women within districts, transport provisions made available.	ANC, delivery & Post Natal care and Health systems strengthening.	Private and Public facilities involved.

UCMB: Uganda Catholic Medical Bureau, WB: World Bank, PNFP: private not-for – profit, NMS: National Medical Stores, JMS: Joint Medical stores, MOH: Ministry of Health, VHT: Village Health teams, PFP: Private for Profit, DHT: District Health Team, SM: Safe Motherhood, USAID: United States Development Agency, CIDA, Canadian International Development agency, DFID: Department of International Development (now UKaid), BTC: Belgian Development agency, STI: Sexually transmitted Diseases, MakSPH: Makerere University School of Public health, MSU:Maries Stopes International-Uganda, GPOBA: Global Partnership for Output-Based Aid, CBOs: Community-Based Organisations, HPI: Health Partners International, ANC: Antenatal Care,

**Source: Authors’ analysis**

The following section elaborates on the key design changes in RBF models summarised in the tables above. The findings are organized around three domains, namely: 1) management and governance structures with a focus on the separation of functions of funder holding, purchasing, regulation and service provision/beneficiary, 2) verification, information and payment systems, and 3) service coverage and population coverage/geographical scope.

### Management and governance functions/structures: separation of functions

All the schemes were externally funded, and donor agencies or their delegated agencies performed fundholding functions. The World Bank has been a major fundholder. International organizations have played major fundholding roles as well. For instance, Baylor Uganda and the Infectious Diseases Institute (IDI) were the fundholders for the SMGL Initiative. Health Partners International (HPI) and Montrose were fundholders for the NuHealth Project. Non-governmental organizations (NGOs) (such as MarieStopes Uganda, Cordaid, Baylor Uganda, HPI & Montrose), business entities (such as PricewaterhouseCoopers) and academic institutions (mainly Makerere University School of Public Health) played prominent project management roles, especially in the demand-side schemes. Progressively, government agencies (albeit more at the sub-national level) were involved mainly in verifying results.

The picture was mixed with regards to the separation of fundholding and purchasing functions. Generally, for all schemes where non-state organizations were contracted to manage the schemes, they performed both fundholding and purchasing functions. The Cordaid scheme was an exception where the purchaser was Jinja Diocesan Health Office while Cordaid retained fundholding functions. Initially, the regulatory function was fused with the purchasing role, so the ‘non-state’ purchaser also regulated the scheme’s implementation. The district health management teams (DHMTs) were incorporated into the later schemes (NuHealth and Cordaid) to perform regulatory functions [19,20]. The SDP also worked very closely with the DHMTs as regulators. Most schemes worked closely with health unit management committees (HUMCs) to reinvigorate the participation of the local leaders and communities in the facilities’ governance.

The later schemes introduced ad-hoc governance structures and platforms to provide lesson sharing opportunities. For example, the Cordaid Project established a multi-stakeholder steering committee (that included technical, political and Jinja Catholic diocesan leaders) to provide oversight. The Cordaid Project also convened annual health assemblies. The NuHealth project also established regional multi-stakeholder structures to monitor the project implementation [25].

The interviewees pointed out that service providers’ autonomy was respected to a large extent for most RBF schemes in as far as the usage of the RBF funds was concerned. However, the respondents reported ‘non-appropriate’ use when the schemes did not specify guidance on fund use. Examples mentioned pertained to the World Bank PBC study and the NuHealth project. The interview respondents highlighted that the Nu-Health Project adopted a ‘hands-off’ management approach where fund utilization was upon the service providers’ discretion. Respondents revealed examples from the World Bank PBC project. One manager used the RBF funds to construct a gate instead of motivating health workers. At the same time, another decided to throw an end of year party.

Some later supply-side models prescribed that a percentage of the funds received ought to be allocated for staff bonuses and the rest for other expenditures [17,20]. For example, under the Cordaid Project, 40% of the bonus had to be assigned to staff bonuses. To ensure that funds were not misused, RBF schemes mandated all facilities to develop business plans. These business plans served as both strategic and operational frameworks to guide the utilization of the facility funds. For example, both the NuHealth and Cordaid project required annual business plans to be updated periodically [26]. Quarterly plans would be extracted from the yearly plan. Another governance element relates to provider autonomy which was reportedly undermined by the service providers’ limited capacity to negotiate with the fundholders on the targets, services to be provided, and payment methods [17]. The fundholders generally dictated the prices with occasional negotiation between both parties.

#### Verification, data management and payment systems

All RBF models established verification systems as a precondition for payments. Recent models had better structured and more regular verification processes. For instance, in the NuHealth and Cordaid projects, monthly quantity verifications were done in addition to quarterly quality assessments [19,25,27]. Respondents appreciated that their payments were more directly linked to their performance. This experience differed greatly from the first World Bank study where verifications were conducted once annually [28].

The nature of indicators verified also changed from complex outcome measures to simple output indicators. In the World Bank PBC study, the indicators were complex with subsequent payments based on previous benchmarks [7]. The indicators were computed in terms of percentage increase from the score at the last assessment. This created the incentive for facilities to under-declare their performance to keep the benchmarks within reach [28].

Community verification and satisfaction surveys were gradually integrated into later RBF models. For instance, the Cordaid project contracted seven community-based organizations (CBOs) to conduct community surveys [19]. Respondents noted that linking patient tracing to facility bonus improved data quality at the facilities.

#### Service coverage and population coverage/geographical scope

Regarding service packages, all demand-side schemes offered packages under maternal and related child health services. Supply-side pilots provided selective packages from the national minimum health-care packages. However, non-communicable diseases were not considered by all the schemes. There is value in using the general lessons in designing the national RBF model in Uganda. Most projects were implemented in the private sector. Only three schemes (SDP, Cordaid pilot, and the SMGL initiative) were implemented in the public sector. The interview narratives indicated that establishing complementary institutions and public sector reforms supporting RBF had not progressed sufficiently. The reasons for the preponderance toward the private sector are elaborated in the subsequent section.

### Reasons for slow national scale-up of RBF in Uganda’s health system

From the synthesis of data, the slow national scale-up (i.e. integration of RBF into national systems as a national program) was attributed to the following attributes of innovations per the DOI, namely, 1) Compatibility, 2) Trialability and 3) Observability. These issues are elaborated on subsequently.

#### Compatibility of RBF with the Ugandan health system and public sector

### Perceived Incompatibility of RBF with the public health system

Perceived incompatibilities with the public health systems provided a major reason for the limited involvement of public sector facilities in earlier RBF schemes. Okal et al. 2013 also highlighted this concern in their assessment of the opportunities and challenges for public sector involvement in the maternal health voucher program in Uganda [29]. They observed that ‘free’ health-care policy in the public sector was incompatible with RBF:

Some district and national level officials also viewed introducing the voucher program in public health facilities, as in conflict with the government policy of providing services at no cost [29].

This issue was reiterated by a district manager

But the (national) policy is that in public facilities, the health services are largely free apart from the private wings. So now as much as they (government) appreciate the benefits of RBF, the (free health care) policy doesn’t fulfil the requirements of the results-based financing where the user also has to contribute (DHO).

The lack of autonomy of the public health facilities to make critical management choices on inputs, especially, to hire and fire health workers and purchase supplies as needed was considered a barrier to the extension of RBF reforms to the public sector. For example, civil servants in Uganda are ‘permanent and pensionable’ with weak performance management systems to align their performance with systemic goals. Constraints arising from health facilities having limited powers to recruit new staff or dismiss underperforming ones were highlighted.

The moment you introduce RBF, the health facilities are meant to be autonomous- to make decisions on what priorities they want to fund, to hire staff, to fire if need be. But our public sector guidelines and standing orders don’t offer the health facilities with this autonomy that we need for RBF to effectively function (DHO).

On the other hand, the government’s decision to grant a monopoly to the National Medical Stores (NMS) as the sole supplier of all medical supplies to government facilities was reported to constrain the RBF implementation in public facilities. Delays in supplies by NMS undermined the facilities’ ability to address stock-outs in supplies promptly. Concern over this issue was prevalent among respondents:

We know that currently, the government has centralized the supply of drugs. But as far as RBF is concerned, we needed to allow … some service providers to identify other drugs suppliers. We appreciate that National Medical Stores (NMS) has improved the availability of medicines, but we are shy to talk about [un]availability of equipment. For example, delivery kits at these facilities. So, if NMS are not providing the equipment and the facilities cannot buy [these] somewhere else, then we are likely not to achieve improvement in all areas (DHO).

### Compatibility of RBF with existing government systems

Many stakeholders highlighted broader systemic constraints to public sector reforms supportive of RBF. The delays in government reforms were attributed to more general governance and political economy issues on further probing. These were mainly a) the fear of loss of control over resource allocation decisions, b) resistance to change from beneficiaries of favourable allocation of budgets, c) political interference in decision-making, d) poor intersectoral planning, e) unsustainability of the RBF reforms and f) concerns over increased accountability demands under RBF.

The interviews revealed a general perception of the relative advantage of the status quo. The fear of loss of control over resource allocation processes in the health sector among some role bearers was reportedly an obstacle to RBF adoption. Several quotes typify these concerns:

Most of our people (in MOH) are unwilling to change. The health system is structured in such a way that there are power centres at various levels, yet the moment you introduce this RBF, the health facilities are meant to be autonomous- to make decisions on what priorities they want to fund, to hire staff, to fire if need be (DHO)

Top leaders controlling input-based financing have not supported RBF. People fear being rendered irrelevant (Academic)

The resource allocation formula for primary health care (PHC) grants to health facilities and districts used by the government favoured certain units/districts. There was a narrative to indicate that this prompted resistance to change from the current financing arrangements:

The allocation formula for PHC funds is based on [health facility] level. But it doesn’t necessarily mean that a health centre 1V generates more and better outputs than a health centre III. So, some of the people who have benefited from that system of allocating resources are reluctant to let go to RBF (DHO).

Inadequate stewardship capacity to steer RBF within the health sector was reported during interviews. Some key informants referred to top government officials’ preference to follow political directives to drive sector actions without regard to the strategic plans. The implication is that the national scale-up of RBF might delay until RBF becomes a priority among the country’s top political leadership. About this, one respondent remarked:

The top (health ministry) management only want to listen to the president. For example, he orders, ‘build a hospital here and a clinic there’; ‘Increase doctors’ pay at the Health centre IV more than others’. How do you pay one person in a team? (Policy-maker, MoH).

Stakeholders pointed out silo-based planning for reforms as another fundamental barrier to the scale-up of RBF in the country. The administrative practices of the government entities in Uganda encourage sectors to plan and work in silos. Respondents observed that engagements between the Ministry of Health and other key ministries relevant to RBF reforms such as local government, public service and finance were suboptimal. The RBF discourse had not permeated beyond the health sector, yet successful implementation required complementary government-wide reforms. One policy-maker at MoH emphasized that ‘*government systems are disjointed to support RBF*’. Several key informants also noted that RBF principles had not taken root in the government planning and budgeting system to facilitate public sector-wide adoption. The interviewees reported that RBF is a financing reform that requires the Ministry of Finance, Planning and Economic Development (MoFPED) to be at centre stage. The Output Budgeting tool (OBT) used by the MOFPED (and within government) at the time of the study was considered an important precursor for a result-based financial system. However, respondents observed that OBT implementation was sub-optimal to be leveraged for RBF scale-up. Public financing for health per capita was also reportedly low and unable to meet the minimum financial requirements needed for optimal sustainability of RBF. Some respondents argued that RBF would require an increase in investments into the health sector, which might not fit into the existing fiscal space. The need for more investments by the government was reportedly a disincentive for the government to adopt RBF.

There is the required minimum investment in terms of money per capita [under RBF]. For instance, about three dollars is required to support the RBF. Our government is reluctant (to adopt RBF) because it is expensive to operate … . So they are reluctant to take it on quickly. (DHO)

Some respondents noted that the mandatory reporting and accountability obligations under RBF created a general hesitancy to adopt new accountability systems associated with RBF reforms:

RBF demands accountability at all levels. Some people are not willing to be accountable and be exposed to scrutiny as under RBF. (DHO)

### Perceived compatibility and relative advantage of RBF in the PNFP sector

Most of the RBF pilots were implemented in the PNFP subsector. The need to support the PNFP sector and the public sector’s incompatibility were reported as the main reasons for this ‘bias’. In general, RBF funding arrangements reportedly fitted well with the financing arrangements in the PNFP subsector. RBF funds were considered complementary to the user fee charges and government subsidies to these facilities. This compatibility of RBF within PNFPs was attributed to the autonomy and flexibility in financial management systems in the private sector as one respondent asserted:

The private sector is more flexible than [the] government; it can navigate [RBF] more easily than [the] government. In government, they prescribe procedures, and if you find a roadblock, you are supposed to stay there. In the private sector, when you find a roadblock, you can start changing direction, and you might find a solution. (Decision Maker, Private sector).

Many respondents emphasized the need to support the private and PNFP subsectors as a complementary incentive to extend RBF to these subsectors. The PNFP sector usually needs external support to obtain the resources required to deliver health services. One respondent pointed out one example of why RBF focused on PNFPs.

There was a need to support PNFP because [the] government was paying better salaries that led to [the] traffic of health workers from PNFP to government (Field staff)

This study found that the government’s partnership with the private sector, especially the PNFPs, was favourable in advancing support for the PNFP sector [30]. Reportedly, the World Bank PBC study was possible because of the existing strong public–private partnership in health in Uganda that provided opportunities for resources to flow from government to the private sector to provide health services [5,28]. The end of the project report emphasized that RBF was started due to the need to establish formal contractual arrangements for the government subsidies to ensure that these funds stimulated the desired results from the PNFP sector:

Giving grants without defining the expected outputs is not likely to promote efficiency, equity, and effectiveness in health care delivery. It is a high time government and private sector, especially PNFP, went into a contractual arrangement, defining the volumes of services and expected outputs (performance-based) to match the funds provided by the government, especially with (an) emphasis on reaching the very poor and other vulnerable groups [28].

Active lobbying by the PNFP actors for RBF was observed at meetings and noted in interviews. For example, one key informant reported that the PNFP subsector collaborated on the Cordaid Pilot because they wanted to demonstrate to the government that their (government-PNFP) relationship could be formalized through performance-based contracts that would explicitly specify each other’s expectations.

We advocated for RBF] because we wanted to demonstrate to [the] government that the (PHC) funds they give us (PNFPs) can be directly linked to performance (Decision maker, Private sector).

### Trialability and observability of RBF

Trialability (related to the possibility of piloting and experimentation) and observability (clarity of results) underscore the role of evidence in shaping scale-up decisions and processes.

#### Inconclusive Evidence on RBF outcomes despite several pilot schemes

Inconclusive evidence on RBF was highlighted as a reason for the low buy-in to RBF. For example, the inconclusive results from the first RBF study influenced people’s perception of RBF negatively. One key informant noted that: ‘*The [PBC] study did not reach a successful conclusion and so did not generate evidence that RBF works. The failure of the first pilot biased certain people against RBF*’ (Project Manager).

The predominant respondents’ views were a) the belief that RBF is not a panacea and b) the complexity of RBF and the need for more information on implementation issues among policy-makers. *‘There is an understanding that RBF is not a magic bullet to all the problems in our system*,’ one Academic remarked.

Some respondents attributed the slow buy-in into RBF to scepticism that RBF may not be the only reform that Uganda’s health sector needs to improve its performance. The respondents emphasized other structural challenges in the health sector that need to be worked on to ensure that the health system is functional enough to start demanding results from health facilities. *‘RBF is seen as a solution without fully understanding the problem,’* remarked one development partner.

Consensus on how to achieve results seemed inadequate among government agencies. One key informant observed that the Ministry of Finance holds a different view on how developments in the health sector should be boosted, and this was not necessarily through RBF:

*‘The Ministry of Finance is interested in increasing value for money and increasing effectiveness and is convinced that its guidelines and planning tools are sufficient. They do not understand the urge to shift to RBF because they think that their approach is how [they] should be giving them the results. But unfortunately, when you evaluate the success of their approach, it almost ends up being business as usual’* (Development Partner).

Several preconditions for the national RBF rollout were highlighted [17] but reportedly not yet in place at the time of this study. These included a careful combination of RBF with other health financing mechanisms (input-based financing, user fees, and insurance schemes) and ensuring minimal levels of functionality of health facilities:

All health facilities need to have a certain minimum level of operational capacity before being taken up in an RBF scheme. RBF cannot solve structural problems but [can] only capitalize on existing productive facilities to improve service delivery capacity with more efficient utilization of the available assets. In other words, productive assets at [the health facility level need to be in place before introducing RBF [17].

Discussion during the 2015 National Consultative Workshop also highlighted gaps in the funding of basic operational costs at every health system level and that RBF would not cover these shortcomings. The workshop participants recommended that the Government of Uganda develop a strategy to uplift the functionality of health facilities before launching RBF [17].

#### Trialability and observability gaps as barriers to buy-in

Inadequate knowledge of RBF implementation was said to enhance RBF complexity and undermine opportunities for national RBF scale-up. The isolated nature of the schemes so far implemented created reservations on national program rollout. One development partner noted that ‘*some RBF schemes have been implemented as vertical standalone projects and not integrated [into existing Systems] … … Patchy pilots all over the country make it difficult to scale up*.’

Some respondents highlighted concerns about the sustainability of RBF. All RBF pilots depended on donor support which scared policy-makers off national RBF adoption. One development partner remarked that ‘*Dependency on donor funding to reward output [is not sustainable]. When delayed or no funding is provided, [this] affects the scale-up of the scheme*’.

RBF pilot schemes were considered very expensive to implement, yet little information was being shared on implementation costs. Stakeholders expressed keenness to know the costs of a national rollout. Yet, little was being provided by the scheme implementers: ‘*Not enough is shared on how much RBF costs … yet it is hard to demonstrate RBF effectiveness. Expensive designs of RBF aren’t sustainable once the funders pull out’* (Academic).

The respondents also felt that service packages’ narrow focus might not provide a complete picture of full-scale RBF implementation costs.

#### Inadequate information sharing

Despite various stakeholder engagement and awareness creation efforts, such as undertaking national and international study visits and organizing national workshops, some respondents felt that buy-in into RBF was still low at the national level.

We *have held [national consultation] workshops, and I think people understand RBF better, but it is hard to change the mindset. However, I believe that after five years, it [RBF] will be embraced*” (Development partner).

The slow progress of RBF was partly attributed to inadequate engagement of key stakeholder such as relevant ministries (health & Finance) and the politicians at the national level. A manager of one of the projects remarked: ‘*Those who have the money, the politicians have not understood it [RBF] … and that is why they are dragging their feet to slow down the scaling-up process*’. Some respondents reported low participation within MOH as well. *‘[RBF has moved] to a small extent citing the two consultative meetings that we held but policymakers were not there yet. There were few people from the Ministry of Health*’, one project manager observed. Within government, unlike local governments, the central-level MoH had been less involved in RBF implementation. For example, it was documented that the PBC study recruited and positioned advisors for RBF in MOH. These advisors left after the RBF pilot [28,31] and went to advise Rwanda on its scale-up process.

Several respondents noted that knowledge sharing has concentrated among researchers, pointing to the ineffective translation of RBF knowledge. ‘*Cross-fertilization of knowledge [on RBF] is only a domain of research institutions like [Makerere University] School of Public health … Sharing knowledge about RBF has not been very optimal*’ (Policy-maker, MoH).

## Discussion

This paper provides a comprehensive description and comparison of the designs of key RBF schemes implemented in Uganda between 2003 and 2015. This study examined four supply-side pilots and three demand-side voucher schemes. The supply-side initiatives mainly targeted the service providers (health facilities). The demand-side targeted the consumers/users of health services primarily. However, there were overlaps between these two categories. The schemes differed to varying degrees in terms of results being secured, target populations, their design and implementation arrangements (e.g. partnerships, performance audits and payments systems).

This study also contributed to bridging the evidence gap on reasons for the slow scale-up of RBF in developing countries by examining RBF schemes implemented in Uganda between 2003 and 2015. RBF reforms introduce changes in institutional arrangements, property rights and incentives for the health system actors [8]. This reality seems to be a major deterrent for buy-in for RBF in Uganda. Scrutiny of the various models indicated progressive efforts towards designing a model appropriate for the Ugandan context. In some cases, the learning agenda was deliberately embedded in the scheme design. For instance, several pilots were experimental in design to generate evidence and knowledge of what could work or not. Some cross-scheme learning was reported. Several lessons had been harnessed over the years to improve RBF integration and sustainability in the national health system. This is vital for implementing complex interventions like RBF characterized by uncertainty and often negative unintended effects [7,8,32]. This observation underscores the value of learning from implementation experiences [33]. The health systems stakeholders should leverage opportunities for cross-fertilization of knowledge on RBF design and implementation emanating from these in-country experiments and experiences.

The study revealed concerns that key stakeholders such as the MOFPED and the Ministry of Local government were not fully engaged in the RBF development processes. Knowledge and implementation experience is concentrated at service provider level and less at system or policy levels. This reflects the implementation space where RBF needed to move to be integrated with the health system. Recent efforts to bring stakeholders together [17,34] were noted. These should continue to improve awareness among key stakeholders. Proponents of RBF should take advantage of the government’s (34,35) ongoing efforts and the regional initiatives [35] to establish results-based management approaches. It is also vital for policy processes to engage policy-makers in knowledge generation through techniques like embedded research.

Key issues to consider improving compatibility and acceptance of RBF include 1) determining the benefit packages reflective of the demographic and epidemiological profiles of the country, 2) distribution of bonuses at the facility level, 3) use of DHMTs to perform verification roles and 4) extending a degree of autonomy to managers at public facilities. Realization of specific objectives such as equity may require adjustments in indicators and bonuses to disadvantaged regions/groups [36,37].

Implementing RBF in public facilities had been slow. Only three schemes (SDP, Cordaid pilot and to some extent, the SMGL initiative) had been implemented in the public sector at the time of the study. Establishing complementary institutions and public sector reforms supportive of RBF had not advanced as expected. This situation was attributed to several reasons, including the general lack of interest in RBF among some policy-makers despite the evidence being provided regarding the benefits of the same. Other grounds for a low buy-in pointed to deficiencies in the pilot designs. These included schemes being perceived as expensive, lack of effective mechanisms to share information, insufficient information, especially on costs, and bias arising from failure of the first schemes. There was also scepticism that RBF could be the magic bullet to address the health systems constraints affecting performance in the country. These stakeholder concerns should not be overlooked as they appear to have undermined the scale-up process.

In this study, we looked at both demand-side and supply-side schemes. The general tendency is to look at these two RBF clusters separately. However, these RBF mechanisms entail many cross-cutting matters that must be considered if they are to succeed. The SDS project’s inclusion provided a unique focus on upstream governance issues often overlooked during RBF designs. The SDS offered lessons on how RBF could incentivize upstream governance entities, which is important for service level RBF schemes’ success.

One limitation of this study is that it concentrated on the major schemes and could have missed some smaller RBF schemes. This paper does not capture the increasing interest in RBF policy deliberations since 2014. Another article in this supplement (Ssengooba et al., 2021) examines these developments. There is enough evidence to indicate that although there was no national model for RBF in Uganda at the time of this study, the various pilots contributed to increased knowledge of RBF and emergent interest in these approaches (Ssengooba et al. 2021).

## Conclusion

The studied RBF schemes were diverse in many attributes. They differed to varying degrees in terms of results being secured, target populations, their design and implementation arrangements (partnerships, performance audits and payments systems). However, there were some convergences and observable trends in certain aspects. In general, these various RBF schemes represented the major efforts between the Government of Uganda, donor and development agencies (e.g. World Bank, DFID and USAID), researchers and academic institutions and other non-governmental organizations in the design, implementation and evaluation of these initiatives.

Progressive learning across schemes and time was a major driver of models’ evolution as modifications of design features were efforts to adopt what works well and address challenges over time. Evidence also shows that lessons were drawn on the use of resources/bonuses, information systems and RBF integration into government systems. Therefore, despite the general conclusion that RBF in Uganda did not attain optimal integration over the 2002–2015 period, we view these schemes as actual opportunities that the country had (and still has for the ongoing experience) to learn and move forward with the RBF agenda. We have also highlighted the systemic level factors that need to be addressed to fully integrate RBF into the national health system.

RBF is a complex intervention. In this study, we advise that Uganda and similar countries should undertake a system fitting of RBF by custom designing its schemes and desist importing ‘best-practices’ from other contexts. We are convinced that understanding linkages between different initiatives and those different models implemented within the same health system yields more robust evidence upon which policies and practices for national scale-up, integration and sustainability of RBF mechanisms can be based on.

## Data Availability

Interview data are available on reasonable request.

## Data deposition

The primary data are not publicly available due to ethical concerns. They contain information that could compromise research participant privacy/consent.

## Paper context

Results-based financing reforms have been implemented in many developing contexts to enhance the performance of health systems and to foster goals such as universal health coverage. This paper is derived from a Uganda case study that was part of a multicountry research initiative entitled ‘Implementation Research: Taking Results-Based Financing from Scheme to System’. The research initiative explored barriers and enablers for the institutionalization of results-based financing as part of national policies.
